# Wide Field-of-view and Broadband Terahertz Beam Steering Based on Gap Plasmon Geodesic Antennas

**DOI:** 10.1038/srep41642

**Published:** 2017-01-30

**Authors:** Kaipeng Liu, Yinghui Guo, Mingbo Pu, Xiaoliang Ma, Xiong Li, Xiangang Luo

**Affiliations:** 1 State Key Laboratory of Optical Technologies on Nano-Fabrication and Micro-Engineering, Institute of Optics and Electronics, Chinese Academy of Sciences, P.O. Box 350, Chengdu 610209, China

## Abstract

Despite a plethora of applications ranging from wireless communications to sensing and spectroscopy, the current terahertz beam steering technologies suffer from tremendous insert loss, stringent control of electric bias, limited scanning angle, relatively complicated configuration and narrow operation bandwidth, preventing further practical application. We propose and demonstrate a conceptually new approach for terahertz beam steering by virtue of gap plasmon geodesic antennas. By adjusting the geometric dimension of the gap plasmon geodesic antennas, all gap plasmon modes add coherently along a peculiar direction that depends on the geodesic mean surface. Consequently, high directive beams are generated through the antenna, whose direction could be changed within a wide-angle range spanning ±45° by lateral motion of the feed. Furthermore, an assembled antenna structure consisting of four-element geodesic antennas array is proposed for full 360° beam steering, which can operate in a broadband range from 0.8 THz to 1.2 THz.

Recent years have seen a plethora of significant advances in terahertz (THz) realm as a consequence of the continuously improving THz sources and detectors^1^. A mature THz technology, however, does not only require THz emitters and detectors but also passive components which can be used to guide or manipulate THz waves, such as lenses^2^, absorbers^3^,^4^ and waveplates^5^. Among the passive components, the need for spatial control of THz beam has become more and more imperative. Flexible direction manipulation has been demonstrated in microwave band based on metamaterials^6^,^7^. The essence of direction manipulation is wavefront engineering, which can be implemented by different phase modulation scheme such as propagation phase retardation^8^,^9^, geometric phase shift^10^,^11^ and circuit-like resonance induced phase abruption^12^,^13^. Recently, a terahertz all-dielectric magnetic mirror formed from an array of silicon cube resonators and simultaneously support electric as well as magnetic dipolar Mie resonances has been proposed^14^, which is capable of engineering a reflected beam’s spatial properties with high efficiency. By controlling the interference between these modes, the amplitude and phase of a reflected wave can be arbitrarily controlled over a sub-wavelength area, which results in the generation of various beam types including vortex and Bessel beams. Nevertheless, most terahertz devices lack the flexibility to dynamically control the electromagnetic responses, which leads to a narrow-band and single-function operation.

Plasmonic antennas integrated with active media enable the dynamic modulation of the optical properties without compromising the device thickness^15^,^16^,^17^,^18^. The capability of dynamically controlling the propagation of THz waves is essential for a variety of applications. For instance, in THz wireless communications, transmission likely rely on point-to-point connections using highly directional beams. Beam steering will enable securing reliable communication paths^19^. Furthermore, the state of the art technique for characterizing samples over a broad frequency range is THz time domain spectroscopy (TDS). Spatially-resolved spectroscopy and imaging applications are based on a relative movement between THz field and samples under test. One possible approach to accomplish such demands is given by a steerable THz emitter^20^.

Although beam steering technologies^17^,^21^,^22^,^23^,^24^,^25^,^26^ have been widely presented in acoustic, microwave, infrared, and visible band, there is only limited well-demonstrated approaches in THz band, encompassing directly changing the laser excitation angles, phased array antenna technique and photoconductive antenna array, etc^27^,^28^,^29^. Nevertheless, there might be certain drawbacks as well, including tremendous insert loss, stringent control of electric bias, limited scanning angle (e.g. no more than 10°), relatively complicated configuration (i.e., required for a large scale antenna array) and narrow operation bandwidth (e.g. some tens of gigahertz), which drastically limits their widespread applications.

Recently, classic curves including centenary curve^30^,^31^, sinusoidal curve^32^ and meandering line^33^,^34^ have been investigated and inspired many exotic optical phenomenon for example vortex beam and Bessel beam generation^30^,^31^, electromagnetic illusion^32^ and polarization manipulation^33^,^34^. Based on a semi-circular curve, we propose a wide field-of-view and broadband terahertz beam steering strategy based on gap plasmon geodesic antenna in this paper. The proposed geodesic antenna constructed by a pair of intersecting parallel plate waveguide (PPWG)^35^,^36^,^37^ supports undistorted terahertz pulse propagation in a broad frequency band. Spherical wavefront radiated from a small feed propagates along their geodesic mean surface. Geometric optics are explored to determinate the geometric parameters of the antenna so that spherical wavefront transform into plane wavefront after through the antenna. Numerical characterizations in both near-field and far-field demonstrate high directive THz beams are generated, whose direction could be changed within ±45° by shifting the feed along the plasmonic antenna. Finally, we show that the scanning range can be further extended to full 360° by a four-element assembled antenna structures and the relative bandwidth exceeds 40% around 1 THz.

## Results

### Structure of gap plasmon geodesic antenna

**Figure 1:**
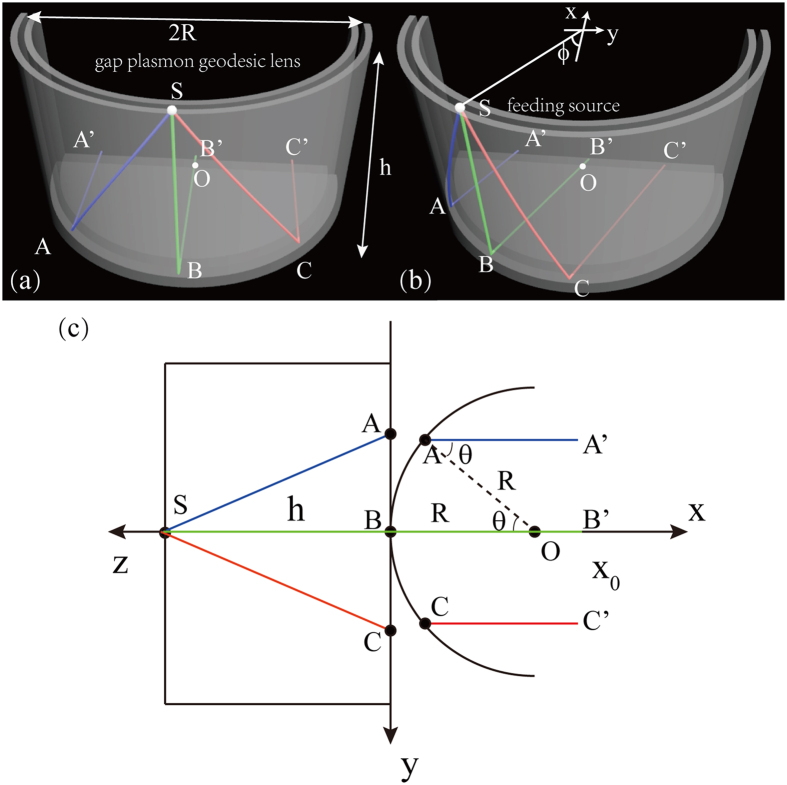
Scheme of proposed gap plasmon geodesic antennas for beam steering in terahertz. (**a**) The feed is located at the center of geodesic lens. (**b**) The feed deviates from the center of geodesic lens. (**c**) Ray trajectories through the plasmonic antenna when it is feed at the geometric center (i.e., *φ* = 0) of the cylinder PPWG.

As shown in Fig. 1(a) and (b), the proposed gap plasmon geodesic antenna for terahertz beam steering is constructed by two right-angle intersecting and conductively joined PPWGs. One is in semi-circular shape extending in the z direction, whose height and radius are defined as *h* and *R*. The other is planar one extending in the *xy* plane. When a small feed with the electric field *E* perpendicular to the conducting plates is disposed at the end of the semi-circular PPWG, it will be coupled to the semi-circular PPWG and generates surface plasmon polaritons (SPPs) on the metal surface. Though SPPs at terahertz show weakly guiding property especially on curved guides due to the huge permittivity of metals, PPWGs will significantly improve the confinement of SPPs because of the strong coupling between the parallel plats, just like the well-known metal-insulator-metal (MIM) waveguide^8^,^38^. Ever since the seminal work on undistorted terahertz pulse propagation in PPWG^36^, this simple but powerful geometry has provided a unique design paradigm and promoted numerous THz applications^35^,^39^. Quite recently, PPWG based leak-wave antennas with spatial (de)multiplexing and focusing ability have been demonstrated in the THz range^37^,^40^.

### Principle of beam steering

Within the frame of geometric optics, different ray trajectories such as those shown in color can be imagined. In accordance with Fermat’s principle, these rays propagate along the geodesic of the mean surface between PPWGs and then radiate from the planer PPWG. With proper height and radius of the semi-circular PPWG, the total path lengths of all rays from the feed to the aperture of antenna will be equal. As a consequence, all the rays add coherently along a peculiar direction, coinciding with the rotation angle *φ* of the small feed. In this circumstance, by shifting the rotation angle *φ* of feed, the direction of THz beams radiate from the antenna could be changed correspondingly.

For the sake of analysis, the semi-circular surface has been flattened and folder in Fig. 1(c) so that geodesic curves on this surface are mapped coplanar with the planar surface^41^. Here, we just consider the case of the small feeding source locating at the geometric centre (i.e., *φ* = 0) of the semi-circular PPWG. Other locations of the feed source enabling different azimuth angles are similar to the case consider here due to the rotational symmetry of the semi-circular surface. In Fig. 1(c), the ray trajectory S-B-B’ represents the axial path-length while ray trajectories S-A-A’ and S-C-C’ represent the off axial path-length. We assume all the radiant rays add coherently in the direction of parallel to the *x* axis after propagating through the antennas and A’, B’, C’ are three points along the contour line of *x* = *x*_0_.

According to the geometric relationship shown in the Fig. 1(c), the path-length of the rays can be calculated as:


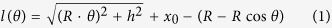


The path-length diffidence between the off-axial ray trajectory and the axial trajectory can be expressed as:


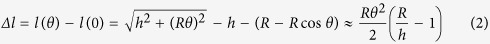


Obviously, when *R* ≈ *h* the path-length diffidence Δ*l* is approximately equal to 0 and all the radiant rays add coherently in the direction of parallel to the *x* axis.

### Radiation and coupling properties of the terahertz feed

Previous investigations mostly employ plano-cylindrical lenses made of high-resistivity silicon to convert the spherical geometry of a radiation beam into a planar one, in order to accommodate the planar geometry of PPWG^35^,^36^,^37^. Nevertheless, this method suffers from the disadvantage of strong Fresnel loss, double pass reflections, and the necessity of time-consuming optics alignment. Alternatively, adiabatic coupling with enhanced coupling efficiency can be realized by virtue of a focusing taper^42^,^43^, which adiabatically reduced the spot size of the free space beam to guarantees low reflection losses at the entrance and exit side of the waveguide. Therefore, beam steering can be realized by changing the focus position of the focusing taper in practice.

**Figure 2:**
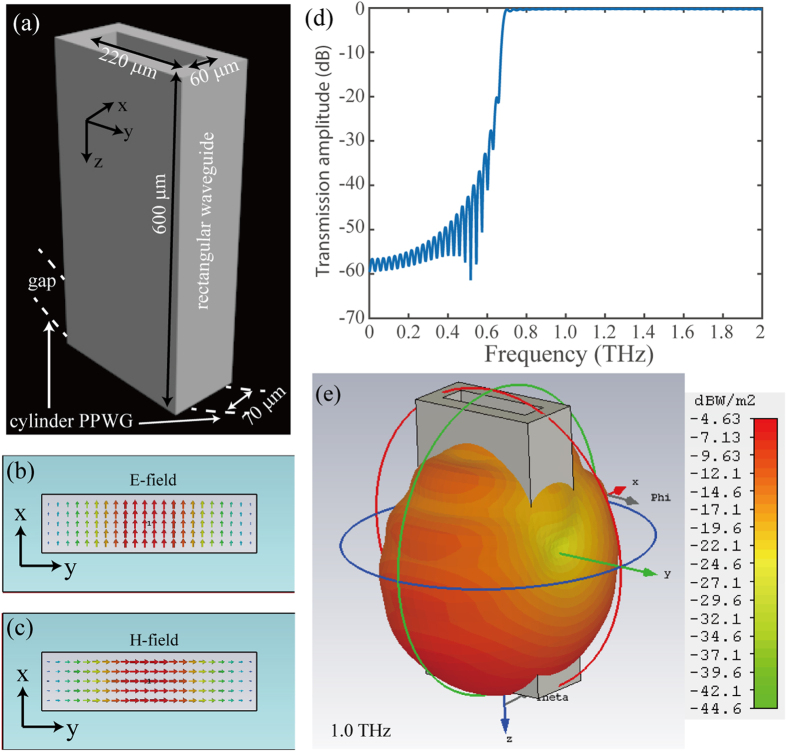
Electromagnetic properties of the THz feed in simulations. (**a**) Geometric structure of the THz feed. (**b**) Electric and (**c**) magnetic field distribution in the rectangular waveguide. (**d**) Normalized transmission amplitude of the rectangular waveguide in the frequency range of 0 THz − 2 THz. (**e**) 3D far field angular distribution of the terahertz feed.

**Figure 3:**
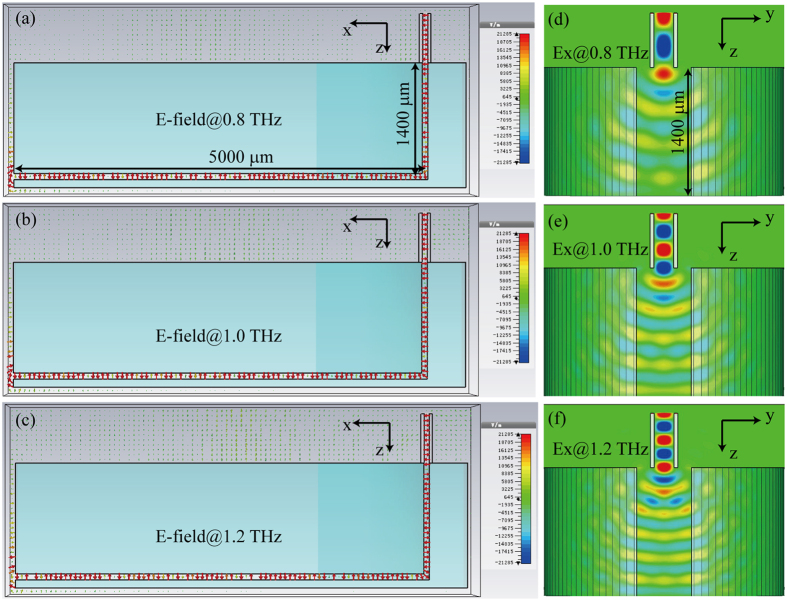
Electric field coupling between the rectangular waveguide and gap plasmon geodesic lens. (**a–c**) Electric field at the cross section of xoz plane at (**a**) 0.8 THz, (**b**) 1.0 THz, and (**c**) 1.2 THz. (**d–f**) Ex field at the cross section of yz plane at (**d**) 0.8 THz, (**e**) 1.0 THz, and (**f**) 1.2 THz.

In this paper, in order to simplify the configuration of adiabatically focusing taper and reduce the computation burden, an analogue of compact THz radiation was realized by a rectangular waveguide with subwavelength aperture, as shown in Fig. 2(a). In order to let most energy is coupled into the PPWG geodesic lens, the rectangular waveguide is located at the top of the cylinder PPWG with their center are aligned. Moreover, the width of the rectangular waveguide is set as 60 μm, a little smaller than the width of PPWG (70 μm), and the length of the rectangular waveguide is set as 220 μm, smaller than the incident wavelength, to ensure the generation of a compact spot size. The transmission properties of the rectangular waveguide were investigated by using commercial software CST Microwave Studios. The electric and magnetic field distribution are shown in Fig. 2(b) and (c), respectively. We can see the supporting electric field in the rectangular waveguide is orthogonal to the gap of PPWG, which behaves like transverse magnetic wave to excite the surface plasmons that propagate along the PPWG. The dispersive propagation characters of the rectangular waveguide was also investigated and shown in Fig. 2(d). One can see that the transmission amplitude is approach to 1 when the incident frequency is higher than 0.7 THz, while it is greatly decreased when the frequency is lower than 0.7 THz since it is not supported by the rectangular waveguide again. The far-field radial pattern and angular distribution of the rectangular waveguide at 1 THz is shown in Fig. 2(e). In order to check the coupling properties between the rectangular waveguide and the gap plasmon geodesic lens (*R* = 1.3 mm, *h* = 1.4 mm), the electric field distributions of different incident frequencies were investigated and shown in Fig. 3. Obviously, most energy is coupled into the geodesic lens and propagates along the gap.

### Near-field characteristics of the gap plasmon geodesic antenna

Subsequently, full model simulations were carried out for the proposed plasmonic geodesic antenna in the frequency range 0 THz − 2.2 THz. The boundary condition, open add space, is the recommended boundary condition for antenna problems. This boundary acts like free space, therefore minimizes reflection. The steady state accuracy limit is set as −30 dB. In order to produce sound and solid results, the mesh lines per wavelength, lower mesh line limit and the mesh line limit ratio are all set to 10. With above mesh controlling, the maximum mesh size is no more than λ/20 of the operation frequency at 1 THz and there are at least 5 mesh grids covering the minimum geometric structure, which offers enough simulation precision^44^.

**Figure 4:**
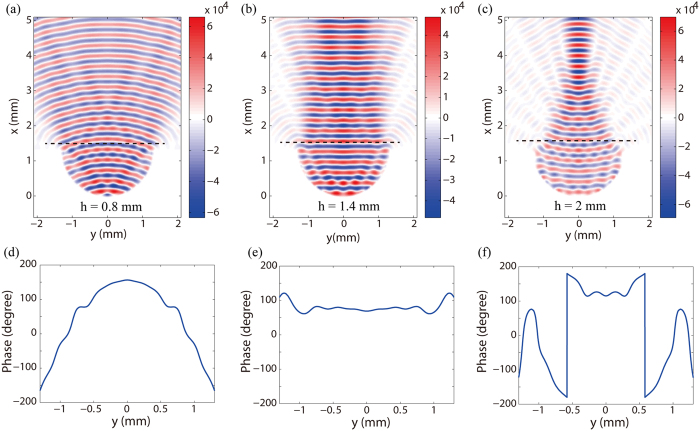
Near-field characteristics of the gap plasmon geodesic lens. Electric field of *E*_z_ component in the planer PPWG with different height of semi-circular PPWG. (**a**) 0.8 mm, (**b**) 1.4 mm and (**c**) 2 mm. (**d,e**) Corresponding phase profile at the cutline x = 1.5 mm indicated in Fig. 4(a–c).

At the frequency of 1 THz, Fig. 4(a–c) show a snapshot of the electric filed component Ez at the mean surface of planar PPWG when a small feed radiates at the center of semi-circular cylinder with different height. We can see when *h* < *R* (e.g., *h* = 0.8 mm) and *h* > *R* (e.g., *h* = 2 mm), the wavefront of radiant wave is divergent and convergent, respectively. Only when *h* ≈ *R* (e.g., *h* = 1.4 mm), we can obtain a plane wavefront propagates in the +*x* direction. Theses simulated results are consistent with the numerical analysis above. Figure 4(d,e) present the corresponding phase profile at the cutline *x* = 1.5 mm indicated in Fig. 4(a–c). Note that, the small amount of ripples present in the figures is due to the diffractions from the edges of the antennas and small reflections from the antenna boundaries.

### Far-field characteristics of the gap plasmon geodesic antenna

**Figure 5:**
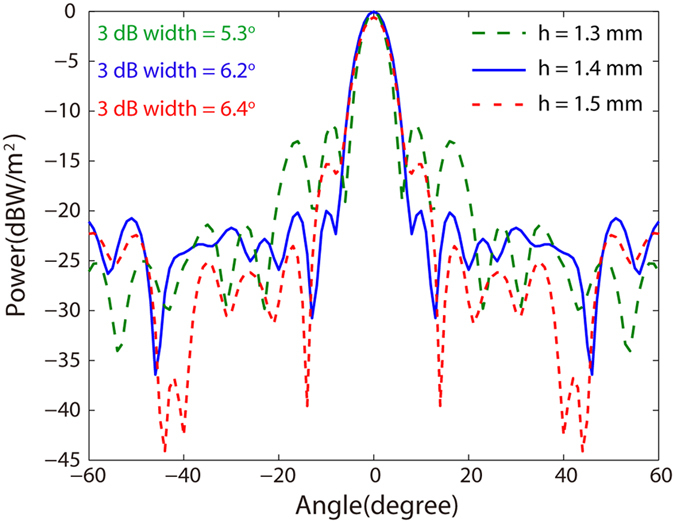
Far-field radiation patterns of the gap plasmon geodesic antenna with different *h*. (**a**) 1.3 mm, (**b**) 1.4 mm and (**c**) 1.5 mm.

Besides the near-field distribution, far-field radiation patterns of the antenna are also checked. As shown in Fig. 5, the radiation angles of the main lobes are all directed towards 0° when h changing from 1.3 mm to 1.5 mm. Especially, when the height is 1.3 mm (*h* = *R*), minimum 3 dB angular width of 5.3° is obtained, which is slightly larger than the theory limit 3.3° due to the diffractions from the edges of the antennas and small reflections from the antenna boundaries. When the cylinder height increases from 1.3 mm to 1.4 mm and 1.5 mm, the 3 dB angular width increases about 1° while the side lobe level decrease from −11.2 dB to −16.5 dB and −14.7 dB, respectively. That is to say, the narrower 3 dB angular width is at a cost of higher side lobe level, just like the well-known super-oscillation phenomenon^45^,^46^. Narrow 3 dB angular width and low side lobe level are pursuit in the design of antennas. Since both the 3 dB angular width and the side lobe level increase with the height of cylinder PPWG, the cylinder PPWG height is fixed at 1.4 mm in the following context so that the 3 dB angular width is only 6.2° and the side lobe level maintain at −16.5 dB.

### Beaming scanning ability of single gap plasmon geodesic antenna

**Figure 6:**
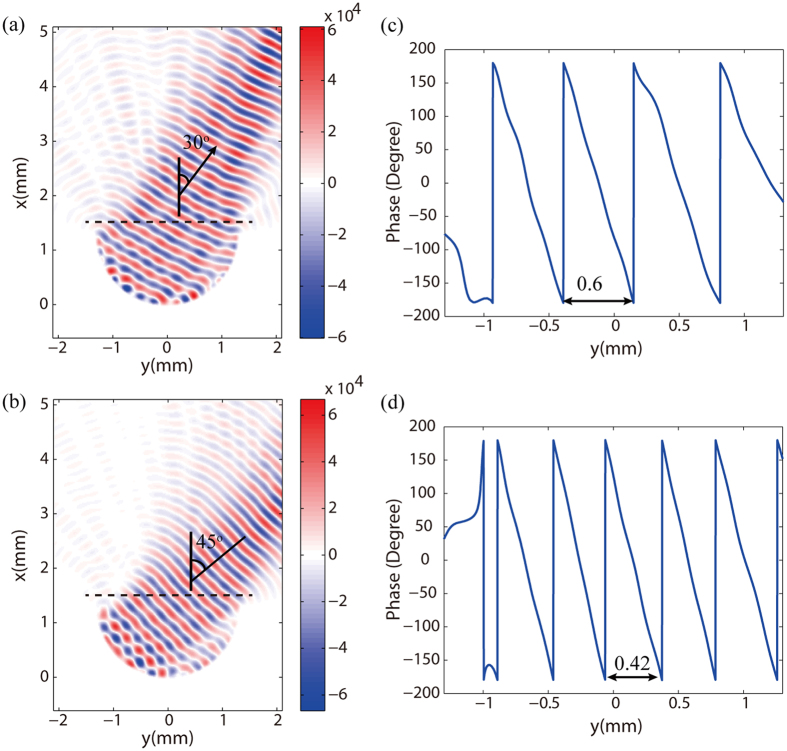
Near-field characteristics of beam steering ability of single gap plasmon geodesic lens. (**a,b**) Electric field of Ez component in the planer PPWG with a rotation angle of 30° and 40° of feed. (**c,d**) Corresponding phase profile of Ez along the black dotted curve in (**a,b**).

**Figure 7:**
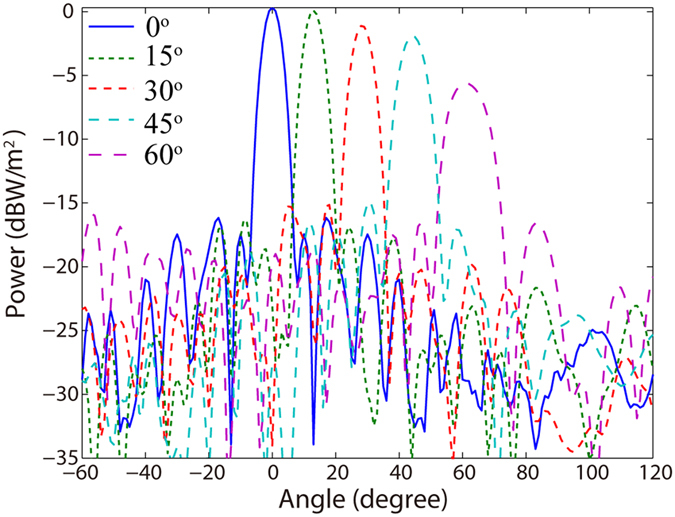
Far-field characteristics of beam steering ability of single gap plasmon geodesic lens. Simulated far-field radiation patterns of the plasmonic antenna with different feed angle *φ*.

Subsequently, we investigate the beam scanning ability of the proposed antenna by the lateral motion of the feed along the semi-circular PPWG. Figure 6(a) and (b) illustrate the near-filed distributions of *E*_*z*_ at the mean surface of planar PPWG when the rotation angle of the feed is 30° and 45°, respectively. It can be seen that the plane wavefront deflect from + *x* direction when the feed rotates along the semi-circular PPWG. In order to estimate the direction of the electric field, the phase profiles along the black dotted line, representing the line of *x*_0_ = 1.5 mm, are also be plotted in Fig. 6(c) and (d). According to the phase gradient, we can calculate the direction of the electric field has an inclined angle of 30° and 45° with +*x* direction, coinciding with the rotation angles of the feed. The far-field radiation patterns of the antenna with main lobes directing towards 0°, 15°, 30°, 45° and 60° are shown in Fig. 7, which indicates the proposed antenna is able to deflect the propagation direction of the THz beam by ±60°. Nevertheless, when the feeding angle exceeds 45°, the beam quality begins to deteriorate due to the decreased peak-power and broadened 3 dB angular width.

### Four-element assembled gap plasmon geodesic antenna array for 360° beam steering

**Figure 8:**
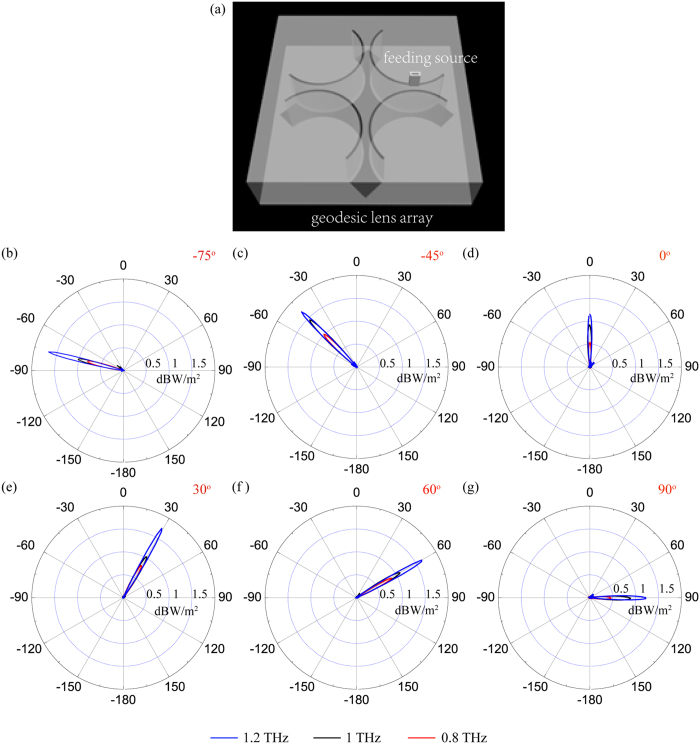
Far-field characteristics of beam steering ability of geodesic lens array. (**a**) A prototype of assembled antenna structure with full 2D scanning ability consisting of four-element antenna. Far-field radiation patterns of the assembled antenna structure at frequencies of 0.8 THz, 1 THz and 1.2 THz. (**b**) −75°, (**c**) −45°, (**d**) 0°, (**e**) 30°, (**f**) 60°, (**g**) 90°.

In order to obtain high quality of directional beam in the whole range of −180° −180°, a prototype of assembled antenna structure composed of four-element antennas with a rotation angle of 90° between them is shown in Fig. 8(a). Due to the 4-fold symmetry of the assembled antenna, each antenna only need steer in ±45° angle with respect to its symmetric axis. Consequently, it is accessible to scan high quality of directional beam in the full plane by shifting the small feed along the track of them. For example, the far-field radiation patterns of above assembled structure with the main lobe deflect towards −75°, −45°, 0°, 30°, 60° and 90° are illustrated in Fig. 8(b–g). In addition, to demonstrate the broadband operation band of the gap plasmon antenna, simulations are performed at frequencies of 0.8 THz, 1.0 THz, and 1.2 THz. The simulation results confirm the gap plasmon antenna perform beam steering well in a frequency range of 0.4 THz. Although this beam steering technique requires mechanical movement of either the feed or the antenna, by adopting multiple feeds at fixed locations, mechanical movement can be avoided but at a cost of discrete scanning manner.

The radiation efficiency of the gap plasmon geodesic antenna including the 4-element case is shown in Table 1. Obviously, the radiation efficiency is higher than 70% for different incident frequency and radiation direction. The small efficiency fluctuation is due to different exaction efficiency of gap plasmon when the feed locates at different positions.

## Discussions

We have shown that by optimizing the geometric parameters of the gap plasmon antenna, where rays are restricted to propagate along a surface midway between PPWG, semi-circular wavefronts can be transformed in planar ones with high directive beams generated in the far field. Beam scanning range exceeding ±45° is realized by shifting the feed along the plasmonic antennas. Full two-dimensional (2D) steering ability has been demonstrated by utilizing assembled plasmonic antennas. The proposed plasmonic antennas can operation in a broad range with relative bandwidth exceeding 40%. The proposed beam steering technology has the advantages of low cost, simple structure, wide scanning angle and broadband operation band, which may find potential applications THz communication, image and spectroscopy where high-gain beam steering capabilities are urgently required. It is undeniable that the propagating of THz-wave in planner PPWG is only confined in one transverse dimension giving rise to divergence loss at long propagation. The two-dimensional (2D) confinement (in combination with dispersion-free THz pulses propagation) can be obtained by using modified PPGW with a tapered and enlarged output aperture, where the guide wave radiates into free space in an adiabatic and high directional fashion. Moreover, we can also add subwavelength grooves/gratings at the output aperture^46^,^47^,^48^ or adding focusing lens^35^,^36^,^37^ at the receiver as done in the previous literatures for beam focusing and collimation.

## Additional Information

**How to cite this article**: Liu, K. *et al*. Wide Field-of-view and Broadband Terahertz Beam Steering Based on Gap Plasmon Geodesic Antennas. *Sci. Rep.* **7**, 41642; doi: 10.1038/srep41642 (2017).

**Publisher's note:** Springer Nature remains neutral with regard to jurisdictional claims in published maps and institutional affiliations.

## Figures and Tables

**Table 1:** The radiation efficiency of the 4-element assembled gap plasmon geodesic antenna.

Angle(°)	0°	±30°	±45°	±60°	±75°	±90°
Fre. (THz)						
0.8	0.80	0.78	0.72	0.77	0.80	0.80
1.0	0.80	0.75	0.73	0.74	0.75	0.81
1.2	0.81	0.73	0.71	0.73	0.76	0.81
